# Mortality and Vital Statistics for the Eleventh Census

**Published:** 1889-09

**Authors:** 


HEALTH PHYSICIAN EDWARD CLARK, of this city, has received the following letter from Dr. John S. Billings, Surgeon United States Army, who has charge of the Mortality and Vital Statistics of the Eleventh Census:

DEPARTMENT OF THE INTERIOR,

CENSUS OFFICE, WASHINGTON, August 21, 1889.

DR. EDWARD CLARK, *City Health Physician*, *Buffalo*, *N. N.*:

DEAR SIR—Having been placed in charge of the vital statistics of the next Census, it is my desire to make them as complete as possible in relation to the death-rates in different parts of the United States, and for this purpose I am desirous of having returns from the City of Buffalo as complete as possible.

The Superintendent of the Census will make a formal application for permission to have copied by clerks, who will be sent for that purpose, the registers of death filed in your office for the census year beginning June 1, 1889, and I sincerely hope that it will be found convenient to grant this request.

A special effort will be made in this Census to obtain the statistics of the mixed bloods between whites and blacks, that is, of mulattoes, quadroons, &c. Under the terms of the law, these are to be specially enumerated and distinguished from the pure whites and pure blacks, and it is highly desirable that we should, as far as possible, in all records of deaths of colored persons, have the information as to whether the decedent was a pure negro or of mixed blood, in order to prepare comparative death-rates for that class. I would therefore respectfully ask of your Board that, if possible, it be distinctly recorded on the death certificate of every colored person dying in Buffalo, between June 1, 1889, and May 31, 1890, whether the decedent was probably a pure negro or of mixed blood, such as mulatto, quadroon, &c.

Do the certificates of death occurring in hospitals, sent to you for registration, contain the street and number from which the patient was sent to the hospital? If not, can you not undertake to obtain this information for all deaths occurring in hospital during the census year, beginning June 1, 1889?

Hoping that you may be able to grant these requests, and will use your best influence with the physicians of the city to have these records made as complete as possible, I remain, Very respectfully,

JOHN S. BILLINGS, *Surgeon Uuited States Army.*

In charge Mortality and Vital Statistics Eleventh Census.

Inasmuch as Dr. Billings has chosen Buffalo as one of the cities whose mortality is to be copied, it is sincerely hoped that all physicians in the city will see to it that all death certificates filed by them are full and complete. The information which he is aiming to obtain relative to the death-rate among the colored races is very important indeed. We have the assurance that the Board of Health will extend to Dr. Billings' representatives every courtesy, and render them all the assistance possible in carrying out this very important work. A careful perusal of the letter will indicate the points upon which Dr. Billings requires special information.

